# Isolation and characterization of *Bacillus* sp. GFP-2, a novel *Bacillus* strain with antimicrobial activities, from Whitespotted bamboo shark intestine

**DOI:** 10.1186/s13568-018-0614-3

**Published:** 2018-05-22

**Authors:** Jia Wu, Guoqiang Xu, Yangyang Jin, Cong Sun, Li Zhou, Guodong Lin, Rong Xu, Ling Wei, Hui Fei, Dan Wang, Jianqing Chen, Zhengbing Lv, Kuancheng Liu

**Affiliations:** 1 College of Life Sciences, Zhejiang Sci-Tech University, Hangzhou, 310018 People’s Republic of China; 2 School Hospital, Zhejiang Sci-Tech University, Hangzhou, 310018 People’s Republic of China; 3 Zhejiang Provincial Key Laboratory of Silkworm Bioreactor and Biomedicine, Hangzhou, 310018 People’s Republic of China

**Keywords:** *Bacillus* sp. GFP-2, Complete genome sequencing, Antimicrobial peptides, Secondary metabolites, Bacteriocins, β-1,3-1,4-Glucanases

## Abstract

The abuse of antibiotics and following rapidly increasing of antibiotic-resistant pathogens is the serious threat to our society. Natural products from microorganism are regarded as the important substitution antimicrobial agents of antibiotics. We isolated a new strain, *Bacillus* sp. GFP-2, from the *Chiloscyllium plagiosum* (Whitespotted bamboo shark) intestine, which showed great inhibitory effects on the growth of both Gram-positive and Gram-negative bacteria. Additionally, the growth of salmon was effectively promoted when fed with inactivated strain GFP-2 as the inhibition agent of pathogenic bacteria. The genes encoding antimicrobial peptides like LCI, YFGAP and hGAPDH and gene clusters for secondary metabolites and bacteriocins, such as difficidin, bacillibactin, bacilysin, surfactin, butirosin, macrolactin, bacillaene, fengycin, lanthipeptides and LCI, were predicted in the genome of *Bacillus* sp. GFP-2, which might be expressed and contribute to the antimicrobial activities of this strain. The gene encoding β-1,3-1,4-glucanase was successfully cloned from the genome and this protein was detected in the culture supernatant of *Bacillus* sp. GFP-2 by the antibody produced in rabbit immunized with the recombinant β-1,3-1,4-glucanase, indicating that this strain could express β-1,3-1,4-glucanase, which might partially contribute to its antimicrobial activities. This study can enhance a better understanding of the mechanism of antimicrobial activities in genus *Bacillus* and provide a useful material for the biotechnology study in antimicrobial agent development.

## Introduction

The studies of antibiotics started with the discovery of penicillin in 1928. With the discoveries of different antibiotics, many effective therapeutic strategies toward incurable or obstinate disease have been developed. However, accompany with the widely usage of antibiotics, the emerging of antibiotic-resistant pathogens is increasing rapidly, suggesting that, without urgent actions, we are heading for a “post-antibiotic era” (Mahlapuu et al. 2016). To solve this threat, many new and non-conventional anti-infective therapies have been developed (or identified) (Czaplewski et al. 2016). Over the past 10 years, many marine natural products were isolated and showed important effects in the aforementioned therapies. Due to the structural diversities of these natural products, researchers have paid high attention to expanding their studies from sponges, corals to microorganisms, among which, marine microorganisms are regarded as major sources of antimicrobial agents (Ng et al. 2015).

Genus *Bacillus* consists of over 300 species. Members of this genus are Gram-positive, aerobic or facultative anaerobic, rod-shaped bacteria with diverse functions. Recently, peptides produced by members of the genus *Bacillus* were shown to have a broad spectrum of antimicrobial activity against pathogenic microbes. Strains in genus *Bacillus* can produce structurally diverse secondary metabolites, which exhibit a wide spectrum of antibiotic activity, but the precise mechanism is still unclear (Li et al. 2012; Sabate and Audisio 2013; Sumi et al. 2015). The antimicrobial peptides (AMPs) produced by *Bacillus* species can be divided into two subgroups based on the synthesis pathway, one of which includes small microbial peptides that are non-ribosomally synthesized by large enzymatic complexes, whereas the second subgroup comprises ribosomally synthesized peptides (Marahiel 1992; Marx et al. 2001; Nakano and Zuber 1990). Besides, some species in *Bacillus*, like *Bacillus macerans*, also contain genes encoding bacterial β-1,3-1,4-glucanases, which belong to the glycosyl hydrolase family 16 (GH16) and can specifically cleave the β-1,4-glycosidic linkage adjacent to 3-*O*-substituted glucopyranose residues. Because of its specific functions in antimicrobial activity, β-1,3-1,4-Glucanases have been explored in several industrial applications.

In this study, we isolated an antimicrobial bacteria strain, *Bacillus* sp. GFP-2, from the *Chiloscyllium plagiosum* (Whitespotted bamboo shark) intestine, which showed inhibitory effects against both Gram-positive strains and Gram-negative strains and could also promote the growth of salmon. The genes encoding antimicrobial peptides and gene clusters for secondary metabolites and bacteriocins, which might, at least partially, account for the antimicrobial activities of this strain, were predicted by complete genome sequencing and analyzing. Furthermore, a novel *β*-*1,3*-*1,4*-*glucanase* gene was successfully cloned from *Bacillus* sp. GFP-2. The recombinant β-1,3-1,4-glucanase, heterogeneously expressed in *Escherichia coli*, was further purified and used to immunize rabbits to produce an antibody, which could specifically detect β-1,3-1,4-glucanase in the protein extract of GFP-2 culture supernatant, further confirming that *Bacillus* sp. GFP-2 could produce β-1,3-1,4-glucanase and might contribute to its antimicrobial activities.

## Materials and methods

### Culture of *Chiloscyllium plagiosum* and isolation of bacteria colony from intestine

Experimental protocols were reviewed and approved by the Animal Ethic Committee of Zhejiang Sci-Tech University. Whitespotted bamboo sharks were raised in glass container containing water with the salinity of between 1.020 and 1.023% at 25 °C. The health condition was monitored every day and healthy ones were used for this experiment. The shark was sacrificed after injection of 25% urethane as the experiment anesthetic and the intestinal contents and mucosal fluid was taken out and diluted in normal saline as the raw bacteria solution. The raw bacteria solution was further inoculated and diluted on Luria broth (LB) agar plates (5 g of yeast extract, 10 g of tryptone, 10 g of NaCl, and 15 g of agar in 1 L ddH_2_O) and cultured at 37 °C for 12 h. Single bacteria colonies were then picked and further cultured in liquid LB culture media (5 g of yeast extract, 10 g of tryptone, and 10 g of NaCl in 1 L ddH_2_O) at 37 °C for 12 h with shaking at 220 rpm and then were harvested by centrifugation at 12,000 rpm for 1 min.

### Identification of strain GFP-2 and phylogenetic analysis

The strain GFP-2 was cultured in liquid LB media at 37 °C for 12 h with shaking at 220 rpm and then was harvested by centrifugation at 12,000 rpm for 1 min. The Genomic DNA was extracted using the method as described (Sun et al. 2016). The 16S rRNA sequence was amplified by PCR using the following primers: (F27) 5′-AGAGTTTGATCATGGCTCAG -3′ and (R1492) 5′-TACGGTTACCTTGTTACGAC-3′, with the extracted DNA as the template. The almost complete 16S rRNA sequence (1515 bp) of strain GFP-2 was identified on EzBiocloud service (Kim et al. 2012). Phylogenetic trees were reconstructed by the neighbor-joining (NJ; Saitou and Nei 1987) methods with the MEGA 5 program package (Tamura et al. 2011). Evolutionary distances were calculated according to the algorithm of the Kimura two-parameter model (Kimura 1980). OrthoANI values and Unweighted Pair Group Method with Arithmetic Mean (UPGMA) trees between strain GFP-2 and closely related strains were analyzed by Orthologous Average Nucleotide Identity Tool (OAT) (Lee et al. 2016).

### Complete genome sequencing and gene prediction

The complete genome was obtained by using Pacbio RSII platform (Pacific Biosciences, CA, USA). After filter and quality control, all clean reads were assembled using the PacBio HGAP Analysis 2.0 and one circular chromosome with 0 gap was constructed for further analysis. Gene prediction was performed using Glimmer v. 3.02 (Delcher et al. 2007), and functions of the gene products were annotated by BLAST+ (Camacho et al. 2009) using NCBI-nr protein database (Coordinators 2017). The rRNA and tRNA genes were identified by using RNAmmer (Lagesen et al. 2007), tRNAscan-SE (Lowe and Eddy 1997) and Rfam (Griffiths-Jones et al. 2003) database. Classification of predicted genes and pathways were analyzed by using COGs (Tatusov et al. 2003) and KEGG (Ogata et al. 1999) databases. In addition, the RAST online service (http://rast.nmpdr.org/) (Aziz et al. 2008) was used for verification of gene prediction, annotation and classification. The genome circle was draw by CGView application (Grant and Stothard 2008). The antimicrobial peptides were predicted by BLAST+ using the antimicrobial peptide database (APD3)(Wang et al. 2016). The gene cluster for secondary metabolites and bacteriocins were predicted with online servers antiSMASH 3.0 (https://antismash.secondarymetabolites.org/) and BAGEL3 (http://bagel.molgenrug.nl/) (van Heel et al. 2013a; Weber et al. 2015).

### Antimicrobial activity assay

The antimicrobial ability of strain GFP-2 was identified with inhibition zone method. In brief, (1) inoculated activated *Bacillus subtilis* BS168 (Gram-positive, OD_600nm_ = 1.0) and *Escherichia coli* TG1 (Gram-negative, OD_600nm_ = 1.0) as target bacteria into LB agar plates with the concentration of 1:100, v/v; (2) put sterilized oxford cup on pre-coating LB agar plates; (3) added 250 μl of supernatant and related thallus precipitate of strains GFP-2 (OD_600nm_ = 2.0), or 250 μl of ampicillin (1 mg/ml) as the positive control, and then cultured the plates at 37 °C for 12 h; (4) measured the inhibition zones.

### Glucan hydrolysis assay

The crude proteins in extracellular metabolites of *Bacillus* sp. GFP-2 were extracted by ammonium sulfate precipitation method from the culture supernatant. Then the crude proteins or recombinant β-1,3-1,4-glucanase was inoculated into the β-1,3-1,4-glucanase identification agar plates (containing 0.2% of glucan, 0.2% of NaNO_3_, 0.1% of K_2_HPO_4_, 0.05% of KCl, 0.05% of MgSO_4_, 0.001% of FeSO_4_, 0.005% of Congo red, and 2% agar) and the plates were incubated at 37 °C for 12 h.

### Salmon feeding assay

The growth promoting ability of strain GFP-2 to salmon (*Oncorhynchus mykiss*) was evaluated by the weight increment and survival rate of salmon with the feed of strain GFP-2 (strain: fodder = 3:100, in wet weight) after 60 days.

### Growth curve analysis

Overnight cultured bacteria were inoculated into 5 ml of liquid LB culture media at the ratio of 1:100, in the presence of control medium, 25 µg/ml ampicillin, or indicated amount of GFP-2 culture media, and cultured at 37 °C with shaking at 220 rpm. 0, 1, 2, 3, 4, 5, 6, 8, 10 and 12 h after the inoculation, the growth of bacteria was monitored using optical density (O.D) method at 600 nm.

### Cloning of *β*-*1,3*-*1,4*-*glucanase* gene and heterogeneously expression in *Escherichia coli* (*E. coli*)

DNA sequence encoding β-1,3-1,4-glucanase was cloned from GFP-2 genome into the expression construct pET-28a(+) by PCR, which was transformed into *E. coli* BL21 (DE3) to express the recombinant β-1,3-1,4-glucanase. The recombinant protein was further purified through Ni-chelating affinity chromatography and stored at − 80 °C for further use.

### Antibody production by immunizing rabbit with the recombinant β-1,3-1,4-glucanase

2.5 kg weight New Zealand rabbits were immunized with 1 mg of recombinant β-1,3-1,4-glucanase, together with 1 ml of Complete Freund’s Adjuvant (CFA), followed by a second immunization with 0.5 mg of recombinant protein and 0.5 ml of Incomplete Freund’s Adjuvant (IFA) after 15 days. 45 days after the first immunization, rabbit serum containing antibody against β-1,3-1,4-glucanase was collected by centrifugation of the blood at 5000 rpm for 15 min.

### Detection of β-1,3-1,4-glucanase in the GFP-2 culture supernatant

The supernatant of strain GFP-2 culture was collected were analyzed by SDS-PAGE and western blot using the aforementioned rabbit serum containing antibody against β-1,3-1,4-glucanase.

### Nucleotide sequence accession number

The complete genome sequence of *Bacillus* sp. GFP-2 was deposited in DDBJ/EMBJ/GenBank under the Accession Number CP021011.

The sequence of β-1,3-1,4-glucanase was deposited in GenBank under the Accession Number BankIt2108772 BSeq#1 MH260376.

## Results

### Isolation of a bacteria strain GFP-2 from shark intestine that could inhibit both Gram-positive and Gram-negative bacteria

**Fig. 1:**
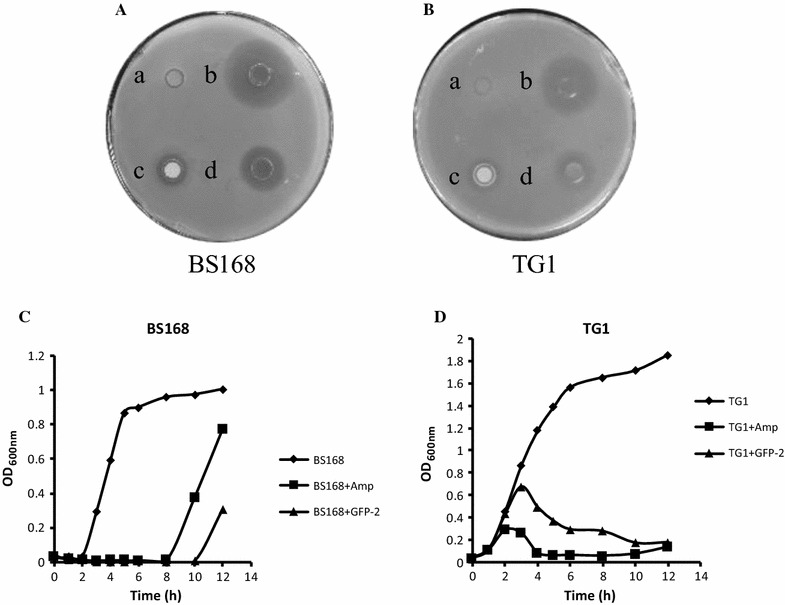
Inhibition of *Bacillus subtilis* BS168 and *Escherichia coli* TG1 by *Bacillus* sp. GFP-2. Activated *Bacillus subtilis* BS168 (Gram-positive, OD_600nm_ = 1.0) (**A**) and *Escherichia coli* TG1 (Gram-negative, OD_600nm_ = 1.0) (**B**) were inoculated as target bacteria into LB agar plates. Sterilized oxford cups were put on pre-coating LB agar plates and 250 μl of LB medium (a, negative control), supernatant (d) and related thallus precipitate (c) of strains GFP-2 (OD_600nm_ = 2.0), or ampicillin (1 mg/ml) (b, positive control), were added onto the plates, and the plates were incubated at 37 °C for 12 h. Overnight cultured BS168 (OD_600nm_ = 3.28) (**C**) and TG1 (OD_600nm_ = 1.86) (**D**) were inoculated, respectively, into 5 ml of liquid LB culture media at the ratio of 1:100, in the presence of control medium (diamond), 25 µg/ml ampicillin (square), or GFP-2 culture media (100 µl for TG1, 500 µl for BS168) (triangle), and cultured at 37 °C with shaking at 220 rpm. 0, 1, 2, 3, 4, 5, 6, 8, 10 and 12 h after the inoculation, the growth of bacteria was monitored using optical density (O.D) method at 600 nm

To investigate the potential antimicrobial products from marine, we screened the bacteria from intestine contents from Whitespotted bamboo sharks, by performing the antimicrobial activity assay. And one of the isolated strains, which was named as GFP-2, showed great inhibition effects against *Bacillus subtilis* BS168, a Gram-positive bacterium, and *Escherichia coli* TG1, a Gram-negative bacterium. As shown in Fig. 1A, both the precipitated bacteria (c) and the culture supernatant (d), especially the latter, formed obvious inhibition zones in the BS168 culture plate, 1.34 cm and 1.70 cm in diameter, respectively, reaching 49.6 and 63.0% of that in the positive control (b, ampicillin). Similarly, both the precipitated bacteria (c) and the culture supernatant (d) of GFP-2 culture inhibited TG1 growth, with 1.28 cm and 1.56 cm inhibition zones in diameter, respectively, 56.4 and 68.7% of that in the positive control (b, ampicillin), as shown in Fig. 1B. Further growth curve analysis showed that, with the presence of GFP-2 culture supernatant, the growth of both BS168 and TG1 were greatly inhibited, similar to that with the presence of 25 µg/ml of ampicillin (Fig. 1C, D). These results indicated that strain GFP-2 can effectively inhibit the growth of Gram-positive and Gram-negative strains, possibly through its secondary metabolites secreted into the supernatant.

### Strain GFP-2 could facility the growth of salmon (*Oncorhynchus mykiss*)

Bacterial infection and the following low survival rate and low specific growth rate greatly impaired the pisciculture. To test if the anti-bacteria activities of GFP-2 could benefit the growth of fishes, we fed salmon (*Oncorhynchus mykiss*) with the Diet 1 (Negative control with normal diet) or Diet 2 (normal diet add with 3% inactivated GFP-2 strain) and estimated the growth performance, such as Gained weight, Specific growth rate (%), Feed conversion ratio and Survival rate (%). As shown in Table 1, after 30 days of feeding with diet 2 containing strain GFP-2, the salmon showed greatly higher gained weight (3.8 fold vs. 1.1 fold) and higher survival rate (79.0% vs. 29.8%) than those with the control diet. These results indicated that the antimicrobial activities of the strain GFP-2 could greatly benefit the growth of fishes and could be used as a new potential antimicrobial agent in the pisciculture.

**Table 1:** Growth performances and feed utilization of the *Oncorhynchus mykiss* fed with different diets: Diet 1 (Negative control with normal diet), Diet 2 (normal diet add with 3% *Bacillus* sp. GFP-2)

	Diet 1	Diet 2
Initial weight (g)	300	300
Final weight (g)	631	1442
Gained weight (g)	331	1142
Specific growth rate (%)	11.03	38.07
Feed conversion ratio	1.27	0.37
Survival rate (%)	29.80	79.00

Final weight and Initial weight were calculated in average; Gained weight = Final weight (g) − Initial weight (g); Specific growth rate = 100* (ln Final weight − ln Initial weight)/Duration of experiment (60 days); Feed conversion ratio = feed given (dried weight)/Gained weight (wet weight); Survival rate (%) = (final fish number/initial fish number) *100

### Strain GFP-2 was identified as a novel strain of *Bacillus*

**Fig. 2:**
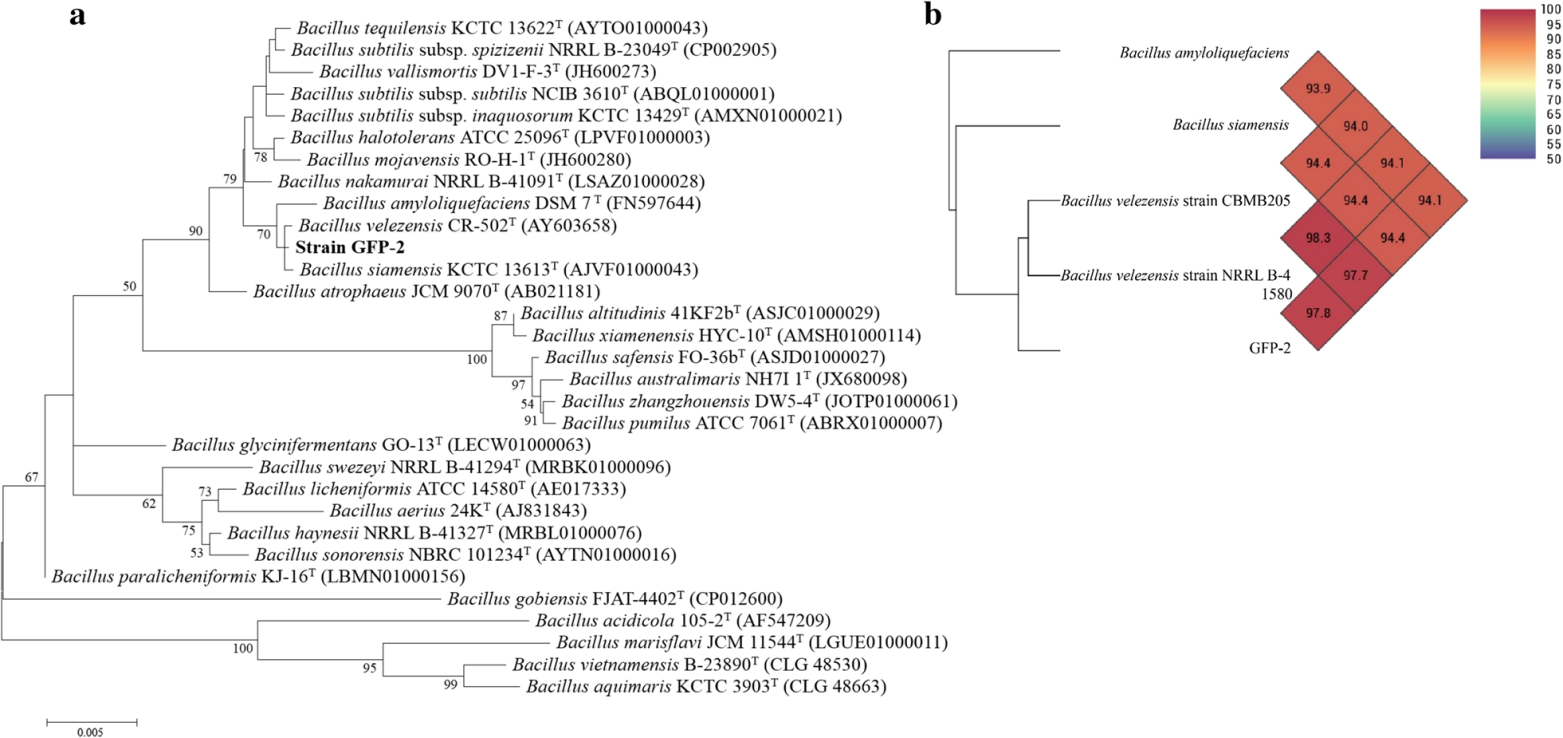
The phylogenetic relationship of *Bacillus* sp. GFP-2 and related strains. The 16S rRNA sequence of *Bacillus* sp. GFP-2 was amplified by PCR and was identified using EzBiocloud service. **a** Phylogenetic trees were reconstructed by the neighbor-joining methods with the MEGA 5 program package. Evolutionary distances were calculated according to the algorithm of the Kimura two-parameter model. **b** OrthoANI values and Unweighted Pair Group Method with Arithmetic Mean (UPGMA) trees between strain GFP-2 and closely related strains were analyzed by Orthologous Average Nucleotide Identity Tool (OAT)

To identify the species of strain GFP-2, 16S rRNA Gene Sequencing was performed and MEGA program was used to establish the phylogenetic tree. As shown in Fig. 2a, strain GFP-2 shared 99.9% of 16S rRNA genetic similarities with *B. siamensis* KCTC 13613^T^ and *B. velezensis* CR-502^T^, 99.7% with *B. nakamurai* NRRL B-41091^T^, *B. amyloliquefaciens* DSM 7^T^ and *B. subtilis* subsp. *subtilis* NCIB 3610^T^, but less than 99.5% with the other *Bacillus* species. And according to this 16S rRNA gene based neighbour-joining tree, strain GFP-2 is located in a separate clade with *B. velezensis* CR-502^T^, *B. amyloliquefaciens* DSM 7^T^ and *B. siamensis* KCTC 13613^T^, indicating their closely phylogenetic relationships (Fig. 2a). To further clarify the phylogenetic relationship of strain GFP-2 with these three species, genome-based ANI values were calculated and UPGMA tree were reconstructed (Fig. 2b). The ANI values of strain GFP-2 with *B. siamensis* KCTC 13613^T^ and *B. amyloliquefaciens* DSM 7^T^ are 94.1 and 94.4, respectively, lower than the species threshold (95–96%) (Goris et al. 2007), and its ANI values with *B. velezensis* CR-502^T^ and CBMB205 are 97.8 and 97.7, respectively, showing that strain GFP-2 is a novel strain of *B. velezensis.* This strain has been uploaded and stored in the China General Microbiological Culture Collection Center (CGMCC No. 13337).

### Genes encoding antimicrobial peptides and gene clusters for secondary metabolites and bacteriocins were predicted in the genome of *Bacillus* sp. GFP-2

**Fig. 3:**
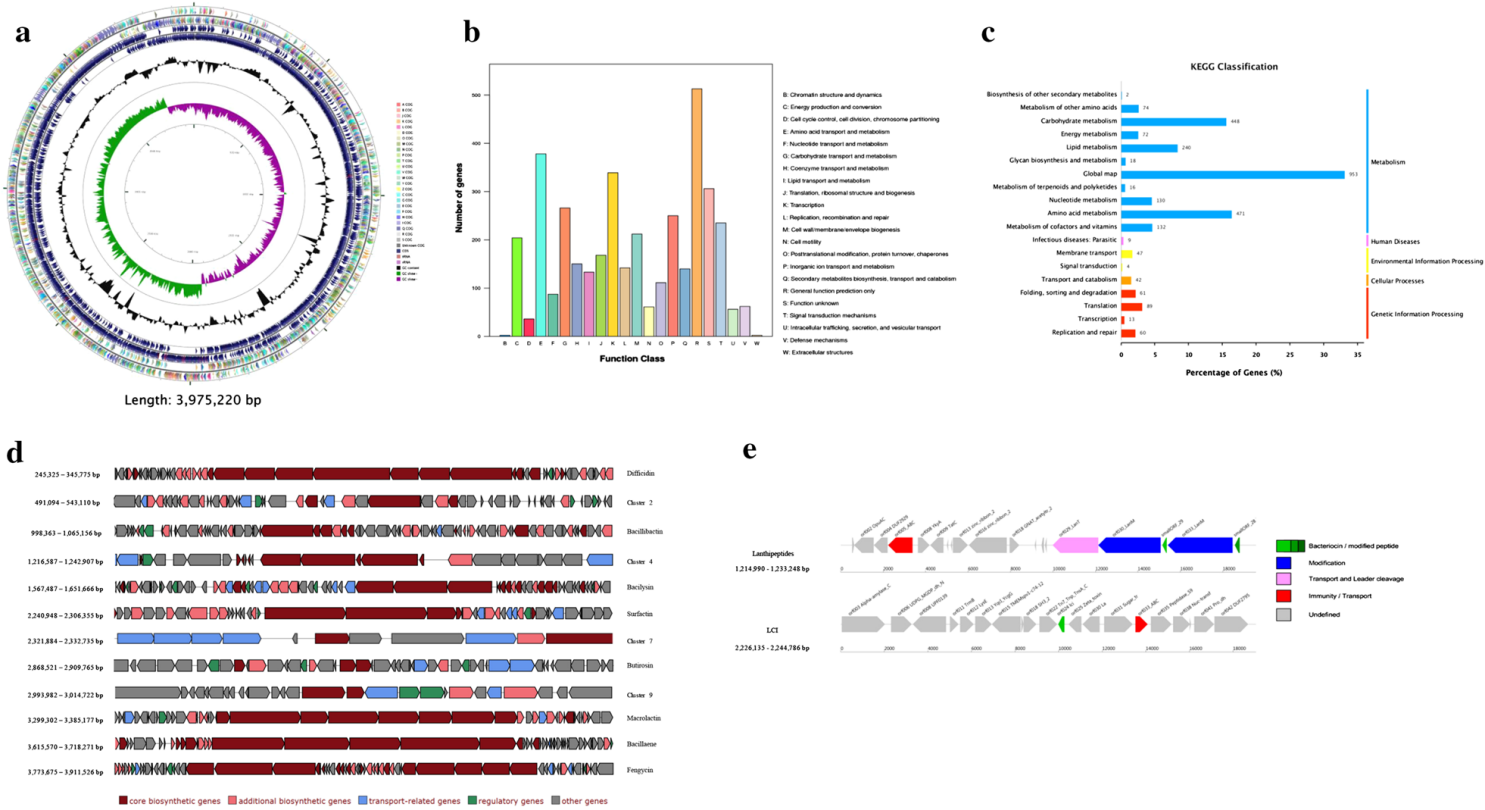
Complete genome sequencing and gene prediction of *Bacillus* sp. GFP-2. The complete genome was obtained by using Pacbio RSII platform (Pacific Biosciences, CA, USA). After filter and quality control, all clean reads were assembled using the PacBio HGAP Analysis 2.0 and one circular chromosome with 0 gap was constructed for further analysis. The genome of *Bacillus* sp. GFP-2 was shown as a circular map: from the outer circle inward, each circle displays information about the genome of forward COG-assigned CDS, reverse COG-assigned CDS, forward CDS, reverse CDS, G + C ratio and GC Skew (**a**). Gene prediction was performed using Glimmer v. 3.02, and functions of the gene products were annotated by BLAST + using NCBI-nr protein database. The rRNA and tRNA genes were identified by using RNAmmer, tRNAscan-SE and Rfam database. Classification of predicted genes and pathways were analyzed by using COGs (**b**) and KEGG (**c**) databases. In addition, the RAST online service (http://rast.nmpdr.org/) was used for verification of gene prediction, annotation and classification. The genome circle was draw by CGView application. The antimicrobial peptides were predicted by BLAST+ using the antimicrobial peptide database (APD3). The gene cluster for secondary metabolites (**d**) and bacteriocins (**e**) were predicted with online servers antiSMASH 3.0 (https://antismash.secondarymetabolites.org/) and BAGEL3 (http://bagel.molgenrug.nl/) (**d**)

As *Bacillus* sp. GFP-2, especially its culture supernatant, showed inhibitory effects on both Gram-positive and Gram-negative strains and could benefit the growth of salmon, we proposed that this strain might produce some metabolites which may play important roles in its antimicrobial activities. So complete gene sequencing was performed to analyze the genome of *Bacillus* sp. GFP-2. The results showed that the genome of strain *Bacillus* sp. GFP-2 (CGMCC 13337) is one 3975,220 bp circular DNA, with the G + C content of 46.4%, in which, a total of 3711 coding sequences (CDS) were predicted, including 19 rRNA and 86 tRNA (Fig. 3a). The summary of general features and statistics of the genome is listed in Tables 2, 3. Among the CDSs, 2893 were classified into clusters of orthologous groups (COGs) categories, the major of which were related to amino acid transport and metabolism (E), transcription (K), carbohydrate transport and metabolism (G), inorganic ion transport and metabolism (P) and signal transduction mechanisms (T) (Fig. 3b). 2881 CDS were classified into KEGG categories, the major of which were related to pathways of global map (33.1%), including biosynthesis of secondary metabolites and metabolic pathways, amino acid metabolism (16.3%) and carbohydrate metabolism (15.6%) (Fig. 3c).

**Table 2:** General features of *Bacillus* sp. GFP-2 and MIGS mandatory information

Items	Descriptions
General features	
Classification	Domain *Bacteria*
	Phylum *Firmicutes*
	Class *Firmibacteria*
	Order *Bacillales*
	Family *Bacillaceae*
	Genus *Bacillus*
Gram strain	Positive
Cell shape	Rod
Color of colonies	White
Temperature	10–40 °C
Optimal NaCl concentration	1%
Optimal pH	7.0
Observed biotic relationship	Free-living
MIGS data	
Investigation type	Bacteria_archaea
Project name	Genome sequence of *Bacillus* sp. GFP-2
Latitude and longitude	Not reported
Depth	Not reported
Geographical location	Not reported
Collection date	Not reported
Environment (biome)	Shark intestine
Number of replicons	1
Estimated size	3,975,220 bp
Source material identifiers	GFP-2 = CGMCC 13337
Trophic level	Chemoorganotroph
Relationship to oxygen	Aerobic
Library reads sequenced	The large smrtbell gdna protocol (Pacific Biosciences, USA)
Sequencing method	A pacbio RS II platform
Assembly data	
Assembly method	De novo assembled using the PacBio hierarchical genome assembly process (HGAP)/Quiver software
Estimated error rate	99.99% (average reference consensus)
Method of calculation	Estimation from HGAP Assembly quality scores
Assembly name	HGAP Assembly version 2 (Pacific Biosciences, USA)

**Table 3:** General features of the genome of *Bacillus* sp. GFP-2

Attribute	Value
Size (bp)	3,975,220
DNA G + C content (%)	46.4
CDSs	3711
CDSs assigned to COGs (percentages)	2893 (78.0%)
CDSs assigned to KEGG (percentages)	2881 (77.6%)
rRNA operon (16S-5S-23S rRNA)	1
tRNAs	86

After blasting the genome sequence with the antimicrobial peptide database (APD3), three hypothetical AMPs were predicted. These three predicted AMPs (peg.781, peg.1300 and peg.2297) were closely related to LCI, YFGAP and hGAPDH, respectively (Table 4). In addition, the prediction of gene clusters for secondary metabolites and bacteriocins were performed and 12 clusters for secondary metabolites (Fig. 3d) and two clusters for bacteriocins, including Lanthipeptides (cluster 1) and LCI (cluster 2), (Fig. 3e) were found and identified. Eight clusters of secondary metabolite have specific functions, including the biosynthesis of difficidin (cluster 1), bacillibactin (cluster 3), bacilysin (cluster 5), surfactin (cluster 6), butirosin (cluster 8), macrolactin (cluster 10), bacillaene (cluster 11) and fengycin (cluster 12) (Fig. 3d). The above-mentioned AMPs, secondary metabolites and bacteriocins have been reported having antimicrobial activities against the bacteria and fungi pathogens, indicating that *Bacillus* sp. GFP-2 may function as antimicrobial agent through producing these molecules.

**Table 4:** Sequences of predicted AMPs by *Bacillus* sp. GFP-2

Predicted AMPs	Sequences	Related AMPs
peg.781	MKFKKVLTGSALSLALLMSAAPAFAASPTASASVENSPISTKADVGINAIKLVQSPDGNFAASFVLNGTKWIFKSKYYDSSKGYWVGIYESVDK	LCI (AP01154)
peg.1300	MKVKVAINGFGRIGRMVFRKAMEDDQIQITAINASYPPETLAHLIKYDTIHGKYDQEVETADDSLIVNGKHIMLFNRRDPRELPWKECGIDIVVEATGKFNSKEKAMSHIEAGAKKVILTAPGKNEDVTIVMGVNEEQFNPDEHVIISNASCTTNCLAPVVKVLDQEFGIESGLMTTVHAYTNDQKNIDNPHKDLRRARACGESIIPTSTGAAKALSLVLPHLKGKLHGLALRVPVPNVSLVDLVCDLKTDVSAEQVNAAFQRAAKTSMYGILDYSDEPLVSSDYNTNSHSAIIDGLTTMVMEDRKVKVLAWYDNEWGYSCRVVDLIRHVAARMKHPSAV	YFGAP (AP02012)
peg.2297	MAVKVGINGFGRIGRNVFRAALNNPEVEVVAVNDLTDANMLAHLLQYDSVHGKLDAEVKVDGSNLVVNGKTIEVSAERDPAKLSWGKQGVEIVVESTGFFTKRADAAKHLEAGAKKVIISAPANEEDITIVMGVNEDKYDAANHHVISNASCTTNCLAPFAKVLNDKFGIKRGMMTTVHSYTNDQQILDLPHKDYRRARAAAENIIPTSTGAAKAVSLVLPELKGKLNGGAMRVPTPNVSLVDLVAELNKDVTAEDVNAALKEAAEGDLKGILGYSEEPLVSGDYNGNANSSTIDALSTMVMEGSMVKVISWYDNESGYSNRVVDLAAYIAKQGL	hGAPDH (AP02017)

### *Bacillus* sp. GFP-2 could express β-1,3-1,4-glucanase

**Fig. 4:**
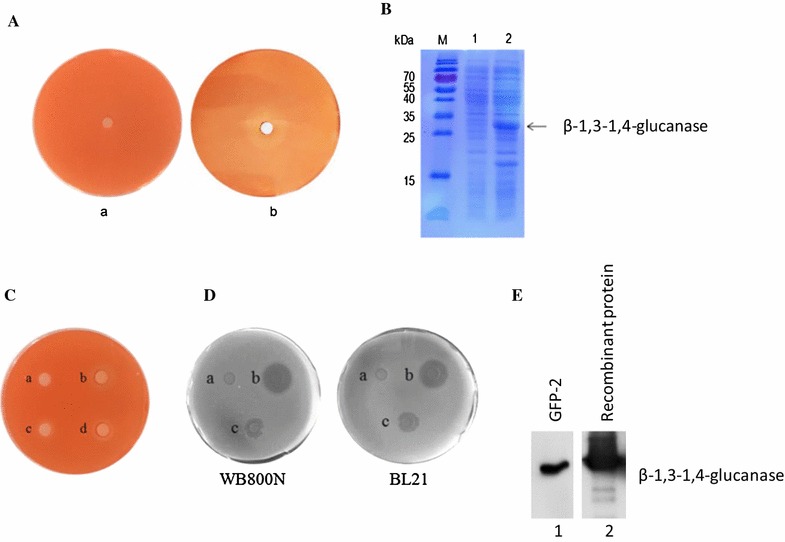
Detection of β-1,3-1,4-glucanase in the culture supernatant of *Bacillus* sp. GFP-2 and conformation of its antimicrobial activities. **A** The crude proteins in extracellular metabolites of *Bacillus* sp. GFP-2 were extracted by ammonium sulfate precipitation method from the culture supernatant. Then 200 μl of Na_3_PO_4_ buffer (a), or the crude proteins (b) was inoculated into the β-1,3-1,4-glucanase identification agar plates (see “Materials and methods”) and the plates were incubated at 37 °C for 12 h. **B** DNA sequence encoding β-1,3-1,4-glucanase was cloned from GFP-2 genome into the expression construct pET-28a(+) by PCR, which was transformed into *E. coli* BL21(DE3) to express the recombinant β-1,3-1,4-glucanase with (line 2) or without (line 1) the stimulation of IPTG. The recombinant protein was further purified through Ni-chelating affinity chromatography and analyzed by Western blot and Coomassie blue staining. **C** 200 μl of elution buffer (a), supernatant of engineered bacteria lysate (b), or protein elution solutions (c and d) were inoculated into the β-1,3-1,4-glucanase identification agar plates (see “Materials and methods”) and the plates were incubated at 37 °C for 12 h. **D** Activated *Bacillus subtilis* WB800 N (left) and *Escherichia coli* BL21 (right) were inoculated as target bacteria into LB agar plates. Sterilized oxford cups were put on pre-coating LB agar plates and 250 μl of LB medium (a, negative control), ampicillin (1 mg/ml) (b, positive control), or purified recombinant β-1,3-1,4-glucanase were added onto the plates, and the plates were incubated at 37 °C for 12 h. **E** GFP-2 culture media (10 μl, line 1) and the recombinant β-1,3-1,4-glucanase (10 μl, 0.913 μg/μl) (line 2) (positive control, see “Materials and methods” for details) were analyzed by SDS-PAGE and western blot using the rabbit serum containing antibody against β-1,3-1,4-glucanase (see “Materials and methods” for details)

According to the complete genome sequencing (Fig. 3a), the gene encoding β-1,3-1,4-glucanase was also found in the genome of *Bacillus* sp. GFP-2. To further confirm if *Bacillus* sp. GFP-2 could encode and express β-1,3-1,4-glucanase, the crude proteins in extracellular metabolites of *Bacillus* sp. GFP-2 were extracted by ammonium sulfate precipitation method and the glucan hydrolysis assay was performed. As shown in Fig. 4A, an obvious hydrolysis zone was observed in the plate inoculated with crude proteins (b), indicating that β-1,3-1,4-glucanase might exist in extracellular metabolites of *Bacillus* sp. GFP-2. Furthermore, the gene sequence encoding β-1,3-1,4-glucanase was successfully cloned from the genome of *Bacillus* sp. GFP-2 into an expressing construct and heterogeneously expressed in *E. coli* (Fig. 4B, line 2). And the purified recombinant β-1,3-1,4-glucanase also had the activity of glucan hydrolysis, as shown in Fig. 4C with obvious hydrolysis zones (c and d). Further study suggested that the purified recombinant β-1,3-1,4-glucanase also had inhibitory effects on the growth of *Bacillus subtilis* WB800N and *Escherichia coli* BL21 (Fig. 4D), confirming its antimicrobial activities. We further immunized rabbits with the recombinant β-1,3-1,4-glucanase to produce an antibody against β-1,3-1,4-glucanase. And β-1,3-1,4-glucanase was specifically detected by this antibody when the crude proteins from *Bacillus* sp. GFP-2 culture supernatant were analyzed by SDS-PAGE and western blot (Fig. 4E, lane 1), further confirming that *Bacillus* sp. GFP-2 indeed could express β-1,3-1,4-glucanase, which may, at least partially, contribute to its antimicrobial activities.

## Discussion

In this study, a new strain *Bacillus* sp. GFP-2 was isolated from the intestine of Whitespotted bamboo shark and could effectively inhibit the growth of Gram-positive and Gram-negative strains, showing around 50–70% inhibition ability of ampicillin. In addition, when fed additionally with strain GFP-2, as the inhibition agents of pathogenic bacteria, the growth of salmon (*Oncorhynchus mykiss*) can be greatly promoted, indicating that strain GFP-2 can effectively inhibit the growth of pathogenic bacteria in the intestine of other fishes besides shark.

After complete genome sequencing, genes encoding AMPs including LCI, YFGAP and hGAPDH-like AMPs and gene clusters for secondary metabolites including difficidin, bacillibactin, bacilysin, surfactin, butirosin, macrolactin, bacillaene and fengycin, and bacteriocins including Lanthipeptides and LCI, were predicted in the genome of strain GFP-2. LCI, an antimicrobial peptide isolated from *Bacillus subtilis*, shows very strong antagonistic activity against the Gram-negative pathogen *Xanthomonas campestris* pv Oryzea of rice leaf-blight disease and Gram-negative bacterium *Pseudomonas solanacearum* PE1 (Gong et al. 2011). YFGAP, a GAPDH-related antimicrobial peptide isolated from the skin of yellowfin tuna, have antimicrobial activity against Gram-positive bacteria, such as *Bacillus subtilis*, *Micrococcus luteus*, and *Streptococcus iniae*. And hGAPDH is a human GAPDH fragment peptide from human placental tissue exhibiting antifungal activity. The existence of LCI, YFGAP, hGAPDH-like AMPs in strain GFP-2 may partially explain its inhibitory effects on Gram negative/positive bacterium. Additionally, YFGAP, hGAPDH-like AMPs are also first predicted in strains of genus *Bacillus.*

Of the predicted secondary metabolites, difficidin and bacilysin have antibacterial activity against *Xanthomonas oryzae* rice pathogens (Wu et al. 2015). Surfactins and fengycins are considered as crucial components in control of plant diseases as they act as antifungal and antibacterial metabolites and have been shown to stimulate plant defense system by inducing systemic resistance (Aleti et al. 2016). Butirosin, an aminoglycoside antimicrobial peptide, is known as the important class of agents, especially against *Mycobacterium tuberculosis* (Kudo and Eguchi 2009). Macrolactin are polyene macrolides containing a 24-membered lactone ring and shows antibiotic effects against vancomycin-resistant *Enterococci* and methicillin-resistant *Staphylococcus aureus* (Kim et al. 2011). Bacillaene was first found to inhibit bacterial protein synthesis, such as, it can selectively inhibit antagonistic fungi of *Termitomyces* and has recently been implicated in interspecies interactions (Muller et al. 2014; Um et al. 2013). Among the two predicted bacteriocins, lanthipeptides are peptides that contain several post-translationally modified amino acid residues and commonly show considerable antimicrobial activity (van Heel et al. 2013b). Meanwhile, the existence of gene cluster for LCI is strong supporting evidence indicating strain GFP-2 can produce LCI. The prediction of these genes in the genome of *Bacillus* sp. GFP-2 indicated that *Bacillus* sp. GFP-2 may function as antimicrobial agent through, at least partially, producing these molecules.

The identification of the gene encoding β-1,3-1,4-glucanase in the genome of strain GFP-2 and the detection of β-1,3-1,4-glucanase in the crude proteins from *Bacillus* sp. GFP-2 culture supernatant confirmed that *Bacillus* sp. GFP-2 indeed could express β-1,3-1,4-glucanase, which may contribute to its antimicrobial activities.

Our study indicated that the newly isolated strain *Bacillus* sp. GFP-2 could act as effective antimicrobial agent by producing AMPs, secondary metabolites and bacteriocins. The study of detailed mechanism is still undergoing. These results can enhance a better understanding of the mechanism of inhibition abilities in genus *Bacillus* and provide a useful tool for biotechnology study in antimicrobial compounds, which may replace abused antibiotics in prevention and clearance of pathogenic bacteria.

## Abbreviations

AMPs: antimicrobial peptides
GAPDH: glyceraldehyde-3-phosphate dehydrogenase
YFGAP: yellowfin tuna glyceraldehyde-3-phosphate dehydrogenase-related antimicrobial peptide
GH16: glycosyl hydrolase family 16
UPGMA: unweighted pair group method with arithmetic mean
OAT: Orthologous Average Nucleotide Identity Tool
O.D: optical density
CFA: complete Freund’s adjuvant
IFA: incomplete Freund’s adjuvant
CDS: coding sequences
COGs: clusters of orthologous groups
